# Magnitude of Anemia and Associated Factors among HIV-Infected Children Receiving Antiretroviral Therapy in Pastoral Community, Ethiopia: A Retrospective Cross-Sectional Study

**DOI:** 10.1155/2020/9643901

**Published:** 2020-09-30

**Authors:** Getahun Fentaw Mulaw, Fatima Ahmed Yesuf, Haftom Temesgen Abebe

**Affiliations:** ^1^Department of Public Health, College of Medical and Health Sciences, Samara University, Samara, Afar, Ethiopia; ^2^Afar Regional Health Bureau, Samara, Afar, Ethiopia; ^3^Department of Biostatistics, School of Public Health, College of Health Sciences, Mekelle University, Mekelle, Ethiopia

## Abstract

**Background:**

The two major comrbidities (anemia and poor nutrition) are common manifestations of HIV-infected children, which threaten their lives. In Ethiopia, there is limited information on the magnitude and factors associated with anemia among HIV-infected children. Thus, this study was aimed to determine the magnitude and factors associated with anemia among HIV-infected children receiving antiretroviral therapy in the Afar region, Ethiopia.

**Methods:**

A cross-sectional retrospective record review was conducted on a sample size of 102 HIV-infected children aged 6 months to < 15 years in selected ART sites of the Afar region from May 1 to 25, 2018. Patient cards from 2009 to 2017 with the required information were considered. A paired sample *t*-test was used to assess whether there is a significant difference in the hemoglobin level before and after the HAART regimen. Multivariable logistic regression was used to determine predictors of anemia. Statistical significance was determined at*p* value < 0.05.

**Result:**

At baseline, 53.9% of study participants were anemic, from which 8.7%, 36.3%, and 9.8% were mild, moderate, and severe, respectively. There was a statistically significant improvement of hemoglobin level following the one-year course of ART treatment from 10.67 ± 1.82 to 11.5 ± 1.5 (*p* value ≤ 0.001): an improvement of 0.83 ± 1.74. Children who were moderately and severely stunted were more than five (AOR = 5.16, 95% CI (1.71, 15.56)) and more than twelve (AOR = 12.45, 95% CI (2.62, 59.21)) times more likely to be anemic than children who were not stunted, respectively. Children whose mothers had not attended ANC follow-up were more than three (AOR = 3.68, 95% CI (1.38, 9.81)) times more likely to be anemic than children whose mothers attended ANC. Children who were in clinical stages 3 and 4 were more than five (AOR = 5.07, 95% CI (1.79, 14.37)) times more likely to be anemic than children who were in clinical stage 1 and 2.

**Conclusion:**

The magnitude of anemia among HIV-infected children was found to be high, which is 53.9%. Nutritional status (stunting), WHO clinical stage, and history of ANC follow-up were the predictors significantly associated with childhood anemia. Thus, interventions for HIV-infected children should consider those factors.

## 1. Background

Anemia is a condition in which the number of red blood cells (and consequently their oxygen-carrying capacity) is insufficient to meet the body's physiologic needs [1]. Numerically, it is defined as a hemoglobin concentration of less than 11 g/dl [2]. Hematological complications have been documented to be the second most common cause of morbidity and mortality in HIV-infected individuals [3]. Anemia is the most common hematological complication of HIV-infection that has a significant impact on the quality of life and clinical outcomes [4, 5].

The estimates of anemia prevalence among HIV-infected individuals in sub-Saharan Africa range from 70–90%, as compared to 20–60% in developed countries [6–8]. The high prevalence of anemia in HIV-infected children in developing countries may be attributed to endemic malnutrition, helminths infections, tuberculosis (TB), malaria, a different spectrum of opportunistic infections, and poor socioeconomic status [1, 9–12]. Studies showed that anemia in children with HIV depends on several factors such as the stage of HIV infection, reduced dietary intake (iron, folic acid, and vitamin B12), altered absorption, concurrent illness such as malaria and hookworm, and other infections [12–14]. It may also be caused by HIV/AIDS itself, HAART, and autoimmune destruction of erythrocytes [10, 12, 15].

Anemia has been a significant predictor of progression to AIDS, and several studies have shown that as hemoglobin levels decrease, the risk of HIV disease progression increases, and it is associated with an increased risk of death in children [16–18]. Anemia is independently associated with decreased quality of life, accelerated disease progression, and increased mortality in HIV-infected individuals [17–20].

Although there are few studies on the magnitude of anemia and associated factors among HIV-infected children in Ethiopia, there are variations in magnitude and associated factors across settings, and also there have been no studies done in the pastoralist regions of the country. Therefore, this study tried to assess the magnitude of anemia and associated factors among HIV-infected children receiving antiretroviral therapy (ART) in selected health institutions of the Afar region.

## 2. Methods

### 2.1. Study Setting and Design

The study was done in health institutions of the Afar region, Ethiopia. An institutional-based retrospective document review was done from May 1 to 25, 2018, on HIV-infected children who had attended ART from 2009 to 2017. The Afar region is administratively divided into five zones and 32 districts, and Samara is the administrative capital of the region.

### 2.2. Sample Size and Sampling Technique

**Figure 1:**
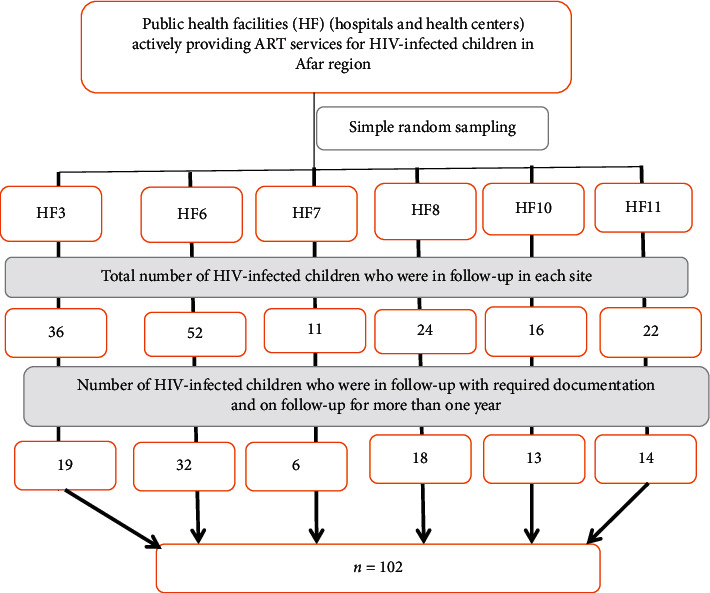
Flow diagram (sampling procedure) for selecting study participants.

There were 25 government health facilities (public hospitals and health centers) actively providing ART services for HIV-infected persons (both adults and children) in the region. Out of these, HIV-infected children, who were on ART, were found only in 12 health facilities. Therefore, from those 12 health facilities, six health facilities (two hospitals and four health centers) were selected randomly. Then, using a universal sampling method, all HIV-infected children in those ART sites/health facilities from the very beginning of the service provision were considered. Then, only 102 HIV-positive children getting ART service had found with the required full documentation. First, children's cards who had received ART drugs for at least one year were selected. Then, the cards were screened based on having full documentation. After all, using the checklist, the required information is recorded (Figure 1).

### 2.3. Study Variables

The potential study variables were classified into four groups. Those were sociodemographic (child age, sex, and residence), nutritional status (anemia status, underweight, wasting, and stunting), HIV infection (viral load, WHO clinical stage, CD4 count, immunosuppression, opportunistic infections, and length of stay on HAART), and childhood illness (pneumonia, measles, malaria, and tuberculosis).

### 2.4. Data Collection Techniques and Tools

Data were collected using a structured questionnaire/checklist based on indicators that can be documented on children's pre- and post-ART cards. Pretesting was done in 5% of the calculated sample size in nonselected ART sites of the region, and then the necessary modifications were done accordingly. Children's anemia status was determined using the WHO's anemia classification based on age. Children aged 6–59 months, 5–11 years, and 12– < 15 years were considered anemic if their hemoglobin level is less than 11 g/dl, 11.5 g/dl, and 12 g/dl, respectively [16, 21].

Children's clinical stage of HIV is also determined based on the WHO clinical stage (stage I, II, III, and IV). Children's immunologic status is also determined using the WHO CD4 classification. CD4% was categorized according to a modified version of the WHO Immunologic Classification for HIV-associated immunodeficiency. It is classified into no evidence of immunosuppression if the CD4 count is 500 (>28%) or more, mild immunosuppression if the CD4 count is between 350 and 499 (20–28%), advanced immunosuppression if the CD4 count is between 200 and 349 (15–20%), and severe immunosuppression if the CD4 count is less than 200 (<15%) [21–23].

The anthropometric status of children was determined by recording height, weight, and children's age from records of ART cards. Then using nutrition software WHO Anthro and WHO AnthroPlus, the nutritional status of children was determined. Height-for-age Z-score (HAZ), weight-for-age Z-score (WAZ), and body mass index- (BMI-) for-age scores of less than −2 SD were considered as stunted, underweight, and wasted, respectively. Those children with a HAZ, WAZ, or BMI score less than −3 SD were considered as severely stunted, underweight, and wasted, respectively [24].

Four health professionals and two public health supervisors were recruited for data collection. The training was given on the data collection process and ethical issues. Continuous supervision and follow-up of the data collectors were done. The collected data were handled and stored carefully and appropriately.

### 2.5. Data Processing and Analysis

Data were checked for incompleteness and inconsistency, edited, cleaned, coded, and entered into EpiData version 3.1, and exported to SPSS version 22 for analysis. Descriptive statistics were used to summarize the characteristics of study participants. A paired sample *t*-test was used to assess whether there is a significant difference in hemoglobin level, BMI, and CD4 count pre- and post-ART. Multivariable logistic regression was used to identify independent predictors of anemia among children receiving antiretroviral therapy. The strength of association was measured through odds ratios at their 95% confidence interval. Predictor variables with a *p* value of <0.25 at bivariable analysis were included in the multivariable logistic regression model and analyzed using the backward stepwise elimination. *p* value <0.05 was used to declare statistical significance.

Multicollinearity was checked using the standard error value. Variables having a standard error value of two or more will be considered having multicollinearity, but no variables were found with it. The percentage of the model that was accurately classified was 67% with Hosmer and Lemeshow goodness-of-fit test (*p* value = 0.605), indicating the model fits well.

## 3. Result

Characteristics of study participants: a total of 102 children participated in this study. From those, 67 (65.7%) children were school age and above, while 35 (34.3%) were preschool age. More than half of children 56 (54.9%) were male (Table 1).

Nutritional status of study participants: at baseline, 29 (28.4%), 56 (54.9%), and 22 (21.6%) of study participants were underweight, stunted, and wasted, respectively (Table 2).

Children's HIV status: dealing with the WHO clinical stage of children, 30 (29.4%) and 20 (19.6%) children were in clinical stage I and IV, respectively. More than half of children (61 (59.8%)) had a history of immunosuppression, and 50 (49%) children had a history of opportunistic infections (Table 3).

Comparison of some characteristics of study subjects pre- and one year post-ART: at baseline, 53.9% of study participants were anemic (8.7% mild, 36.3% moderate, and 9.8% severe), while after a year course of HAART therapy, 46% of children were anemic (23% mild, 18% moderate, and 5% severe). At presentation, before the initiation of HAART, out of all study participants, 28.4% of children were underweight, 54.9% were stunted, and 21.6% were wasted (Table 4).

There was a statistically significant improvement in the hemoglobin level following the one-year course of ART treatment from 10.67 ± 1.82 to 11.5 ± 1.5 (*p* value ≤0.001): an improvement of 0.83 ± 1.74. There is also a statistical improvement of CD4 count from 397.8 ± 233.9 to 568.3 ± 326.9 (*p* value ≤0.001): an improvement of 170.5 + 171.4 (Table 5).

Factors associated with anemia status: in this study, the magnitude of anemia at baseline was found to be 53.9% (95% CI (44.1, 63.7)). Children who were moderately and severely stunted were more than five (AOR = 5.16, 95% CI (1.71, 15.56)) and more than twelve (AOR = 12.45, 95%CI (2.62, 59.21)) times more likely to be anemic than children who were not stunted, respectively. Children whose mothers had not attended ANC follow-up for the index child were more than three and a half (AOR = 3.68, 95% CI (1.38, 9.81)) times more likely to be anemic than children whose mothers attended ANC. Children who were in clinical stage 3 and 4 were more than five (AOR = 5.07, 95% CI (1.79, 14.37)) times more likely to be anemic than children who were in clinical stage 1 and 2 (Table 6).

## 4. Discussion

This study was aimed to identify the magnitude of anemia and its associated factors among HIV-infected children in pastoralist settings (Afar region), northeast part of Ethiopia. According to this study, the magnitude of anemia among HIV-positive children was found to be 53.9%, which is important public health significance. This is slightly lower than the reports of studies done among HIV-positive children in Harer [25] and India [7], which were 54.4% and 66.0%, respectively. On the other hand, this finding is higher than the studies done among HIV-positive children in Gondar [13], Addis Ababa [26], and Uganda [27]. This difference might be due to the age differences and study design used and other sociodemographic characteristics. The finding of this study is also in line with EDHS 2005, 2011, and 2016 reports, in which the prevalence of anemia among non-HIV-infected children aged 6–59 months is 53%, 44%, and 57%, respectively [28].

Even though, there is a statistically significant improvement in the hemoglobin level from baseline, the prevalence after one-year course of ART is still high (42.1%). This could be as our final model shows as the WHO clinical stage is one factor for anemia (other duration goes the likely to progress into the next stage might be high). The other is the infection by itself decreases the appetite, altered absorption, concurrent illness such as malaria and hookworm, and other infections [12–14].

In this study, anemia was independently associated with the nutritional status of children (specifically stunting), WHO clinical stage, and history of ANC follow-up. Children who were stunted were more likely to be anemic than children who were not stunted. Other studies that had done in Ethiopia [25], India [7, 29], and Uganda [27] support this finding. HIV-infected children were likely to have increased nutritional requirements, poor appetite, and reduced intake due to illness and socioeconomic factors [30]. The WHO recommends a 10% increase in energy intake for asymptomatic HIV-infected children, with further increases of 20 to 30% and 50 to 100% for those children who are symptomatic and experiencing weight loss, respectively [30]. Nutritional deficiencies may prone the child to opportunistic infections, and parasitic infestations may result in increased losses, and the presence of chronic infection may depress erythropoiesis [7, 31]. The other possible explanation could be the concomitant occurrence of stunting and anemia due to diminished requirements of micronutrients [32–34].

This study also showed that children whose mothers did not attend ANC follow-up were more likely to be anemic than children whose mothers attended ANC. This can be explained by the fact that during ANC follow-up, a mother could use prevention of mother-to-child transmission (PMTCT) services [35, 36], by which viral load might be decreased and the effective use of HAART might reduce the progression to advanced HIV/AIDS stage [37]. The other possible reason could be that during ANC follow-up, a mother could get nutritional counseling services, which prevents the occurrence of micronutrient deficiencies like iron and vitamin A.

Lastly, children who were in clinical stage 3 and 4 were more likely to be anemic than children who were in clinical stage 1 and 2. The finding of this study is in line with studies done in Ethiopia [25] and India [7]. The possible explanation for this could be that advanced HIV disease may be associated with deficiencies of other micronutrients in children, such as vitamin A, which is thought to have a role in erythropoiesis and iron transport [38]. The other reason can be as HIV progress to advanced stage, the presence of opportunistic infections could further compound anemia [39].

## 5. Conclusion

This study concludes that the magnitude of anemia among HIV-infected children was found to be high, which is 53.9%. Stunting, WHO clinical stage, and history of ANC follow-up were the predictors significantly associated with childhood anemia. There is also a statistically significant improvement in the hemoglobin level and CD4 count following the one-year course of HAART treatment. Thus, interventions for HIV-positive children should consider those factors.

## Figures and Tables

**Table 1:** Characteristics of the study participants by anemia status of HIV-infected children aged 6 months to 15 years, in the Afar region, Ethiopia, 2018.

Variable	Category	Anemia status			*p* value
		Yes, *n* (%)	No, *n* (%)	Total, *n* (%)	
Child age	Preschool age (<7 years)	17 (30.9)	18 (38.3)	35 (34.3)	0.433
	School age and above (≥7 years)	38 (69.1)	29 (61.7)	67 (65.7)	
Child sex	Male	25 (45.5)	31 (66)	56 (54.9)	0.038
	Female	30 (54.5)	16 (34)	46 (45.1)	
Residence	Rural	45 (81.8)	36 (76.6)	81 (79.4)	0.516
	Urban	10 (18.2)	11 (23.4)	21 (20.6)	

**Table 2:** Pre-ART nutritional status of study participants by anemia status of HIV-infected children aged 6 months to 15 years, in the Afar region, Ethiopia, 2018.

Variable	Category	Anemia status			*p* value
		Yes, *n* (%)	No, *n* (%)	Total, *n* (%)	
Underweight	Yes	18 (32.7)	11 (23.4)	29 (28.4)	0.298
	No	37 (67.3)	36 (76.6)	73 (71.6)	
Underweight classification (*n* = 29)	Moderate	11 (20)	8 (17)	19 (18.6)	0.411^*∗*^
	Severe	7 (12.7)	3 (6.4)	10 (9.8)	
Stunting	Yes	40 (72.7)	16 (34)	56 (54.9)	<0.001
	No	15 (27.3)	31 (66)	46 (45.1)	
Stunting classification (*n* = 56)	Moderate	25 (45.4)	13 (27.7)	38 (37.2)	0.149^*∗*^
	Severe	15 (27.3)	3 (6.4)	18 (17.7)	
Wasting	Yes	12 (21.8)	10 (21.3)	22 (21.6)	0.947
	No	43 (78.2)	37 (78.7)	80 (78.4)	
Wasting classification (*n* = 22)	Moderate	8 (14.5)	8 (17)	16 (15.6)	0.417^*∗*^
	Severe	4 (7.3)	2 (4.3)	6 (5.8)	

Moderate = weight-for-age, height-for-age, and weight-for-height *Z*-score < −2SD; severe = *Z*-score < −3SD. ^*∗*^Fisher's exact test.

**Table 3:** Children's HIV status by anemia status of HIV-infected children aged 6 months to 15 years, in the Afar region, Ethiopia, 2018.

Variables	Category	Anemia status			*p* value
		Yes, *n* (%)	No, *n* (%)	Total, *n* (%)	
WHO clinical stage	I	13 (23.6)	17 (36.2)	30 (29.4)	0.011
	II	8 (14.5)	16 (34)	24 (23.5)	
	III	19 (34.5)	9 (19.1)	28 (27.5)	
	IV	15 (27.3)	5 (10.6)	20 (19.6)	
Immunosuppression	Yes	40 (72.7)	21 (44.7)	61 (59.8)	0.004
	No	15 (27.3)	26 (55.3)	41 (40.2)	
Immunosuppression grade (*n* = 61)	Mild	13 (23.6)	13 (27.7)	26 (25.5)	0.044
	Advanced	13 (23.6)	6 (12.8)	19 (18.6)	
	Severe	14 (25.5)	2 (4.2)	16 (15.7)	
Opportunistic infection	Yes	33 (60)	17 (36.2)	50 (49)	0.016
	No	22 (40)	30 (63.8)	52 (51)	
TB	Yes	8 (14.5)	4 (8.5)	12 (11.8)	0.346
	No	47 (85.5)	43 (91.5)	90 (88.2)	
Childhood illness	Yes	33 (60)	25 (53.2)	58 (56.9)	0.489
	No	22 (40)	22 (46.8)	44 (43.1)	
Duration on HAART	<2 years	6 (10.9)	8 (17)	14 (13.7)	0.565
	2–5 years	18 (32.7)	12 (25.5)	30 (29.4)	
	>5 years	31 (56.4)	27 (57.5)	58 (56.9)	
ANC follow-up for the index child	Yes	16 (29.1)	29 (61.7)	45 (44.1)	<0.001
	No	39 (70.9)	18 (38.3)	57 (55.9)	

**Table 4:** Comparison of selected variables using Pearson's chi-square test before the initiation of ART and one year after the initiation of ART regimen.

Variable	Category	At baseline (pre-ART)	After one-year course of HAART treatment	Pearson's chi-square
Anemia	Yes	55 (53.9)	42 (41.2)	0.015
	No	47 (46.1)	60 (58.8)	
Stunting	Yes	56 (54.9)	53 (48)	<0.001
	No	46 (45.1)	49 (52)	
Wasting	Yes	22 (21.6)	18 (17.6)	0.003
	No	80 (78.4)	84 (82.4)	
Underweight	Yes	29 (28.4)	22 (21.6)	<0.001
	No	73 (71.6)	80 (78.4)	

**Table 5:** Comparison of selected variables using paired sample *t*-test before the initiation of ART and one year after the initiation of ART regimen.

Variable	Pre-ART		Post-ART		Paired sample *t*-test				
	Mean	SD^*∗*^	Mean	SD	Mean difference	SD	*t*-value	Df^*∗∗*^	*p* value
Hemoglobin level	10.67	1.82	11.50	1.50	−0.83	1.74	−4.810	101	<0.001
CD4 count	397.83	233.99	568.34	326.90	−170.51	171.39	−10.047	101	<0.001
BMI	14.73	1.38	15.23	1.46	−0.50	1.11	−4.545	101	<0.001

^*∗*^Standard deviation; ^*∗∗*^degree of freedom.

**Table 6:** Factors associated with anemia status of HIV-infected children aged 6 months to 15 years, in the Afar region, Ethiopia, 2018.

Variables/category	Anemia status		COR (95% CI)	AOR (95% CI)
	Yes, *n* (%)	No, *n* (%)		
Sex				
Male	25 (45.5)	31 (66)	1	
Female	30 (54.5)	16 (34)	2.32 (1.04, 5.19)	
Stunting				
No	15 (27.3)	31 (66)	1	1
Moderate	25 (45.4)	13 (27.7)	3.97 (1.60, 9.88)	5.16 (1.71, 15.56)
Severe	15 (27.3)	3 (6.4)	10.33 (2.59, 41.25)	12.45 (2.62, 59.21)
ANC follow-up for the index child				
Yes	16 (29.1)	29 (61.7)	1	1
No	39 (70.9)	18 (38.3)	3.92 (1.72, 8.98)	3.68 (1.38, 9.81)
Evidence of immunosuppression				
No	15 (27.3)	26 (55.3)	1	—
Yes	40 (72.7)	21 (44.7)	3.30 (1.44, 7.54)	—
WHO clinical stage				
Stage 1 and 2	21 (38.2)	33 (70.2)	1	1
Stage 3 and 4	34 (61.8)	14 (29.8)	3.81 (1.66, 8.74)	5.07 (1.79, 14.37)
Opportunistic infections^*∗*^				
No	22 (40)	30 (63.8)	1	—
Yes	33 (60)	17 (36.2)	2.64 (1.18, 5.91)	—

^*∗*^Pulmonary TB, candidiasis, otitis media, toxoplasmosis, pneumocystis carinii pneumonia, cytomegalovirus, and herpes simplex virus were the common opportunistic infections seen in children.

## Data Availability

The data used to support the findings of this study are available from the corresponding author upon request.

## Abbreviations

AOR: Adjusted odds ratio
ART: Antiretroviral therapy
CBC: Complete blood count
CI: Confidence interval
EpiData: Epidemiological information
HAART: Highly active antiretroviral therapy
HIV: Human immunodeficiency virus
MUAC: Mid-upper arm circumference
LZA: Length-for-age Z-score
SPSS: Statistical Package for Social Science
UNICEF: United Nations Children's Fund
WHO: World Health Organization.
